# Type 17 T-cell subset divergence in hidradenitis suppurativa versus psoriasis: a comparative single-cell transcriptomic analysis

**DOI:** 10.3389/fimmu.2026.1835460

**Published:** 2026-08-14

**Authors:** Nayoung Park, Jongeun Lee, Dayeon Kim, Darshna Rambhia, Jaebum Kim, Wei Zhou, Junyue Cao, James G. Krueger, Younhee Ko, Jaehwan Kim

**Affiliations:** 1 Department of Biomedical Science and Engineering, Konkuk University, Seoul, Republic of Korea; 2 Division of Biomedical Engineering, Hankuk University of Foreign Studies, Kyoungki-do, Republic of Korea; 3 Laboratory for Investigative Dermatology, The Rockefeller University, New York, NY, United States; 4 Laboratory of Single-cell Genomics and Population Dynamics, The Rockefeller University, New York, NY, United States; 5 Department of Dermatology, University of California, Davis, Sacramento, CA, United States; 6 Dermatology Section, Veterans Affairs Northern California Health Care System, Mather, CA, United States

**Keywords:** hidradenitis suppurativa, psoriasis, regulatory T-cells, single-cell RNA sequencing, type 17 T-cells

## Abstract

**Background:**

IL-17-producing Type 17 T (T17) cells are central drivers of inflammation in both psoriasis and hidradenitis suppurativa (HS), as evidenced by the clinical efficacy of IL-17-targeting therapies. However, therapeutic responses differ substantially between diseases, raising the possibility that the composition and regulation of T17 states are disease context dependent.

**Objective:**

To define disease-specific T17 subset composition and regulatory T-cell (Treg) transcriptional programs in psoriasis and HS at single-cell resolution, and to generate hypothesis framework regarding their potential relationship to differential therapeutic responses.

**Methods:**

We performed integrated single-cell RNA sequencing (scRNA-seq) analysis of lesional skin from psoriasis (n=20), HS (n=8), atopic dermatitis (AD; n=4), and healthy controls (n=19). T17 subsets were defined based on *IL17A, IL17F, IFNG*, and *IL10* expression, and *FOXP3* expression was used to identify Tregs. Comparative transcriptomic analyses were conducted across diseases to characterize subset composition and functional gene programs.

**Results:**

Psoriasis lesions were enriched in *IL17A*^+^*IFNG*^+^ T17 cells exhibiting cytotoxic and tissue-resident memory signatures, whereas this subset was markedly reduced in HS (19.83% vs 1.27%). In contrast, HS lesions showed expansion of *IL17A*^+^*IL17F*^+^ and *IL17F*^+^*IL10*^-^ T17 subsets, with the *IL17A*^+^*IL17F*^+^ subset demonstrating enhanced inflammatory (*TNF, CSF2, IL21, IL1B, CSF3*) and proliferative (*MKI67*) programs, along with increased *IL1R1* and *OX40* expression. Tregs in both diseases showed reduced *IL10* and immunoregulatory markers; however, HS Tregs uniquely expressed proinflammatory mediators including *IL17A, IL17F, CXCL13, IL1B*, and *CSF3*, indicating a more inflammatory transcriptional state. Dual immunohistochemistry identified cells showing nuclear FOXP3 staining together with IL-1β, IL-17A, or IL-17F protein staining in HS lesions.

**Conclusion:**

These findings reveal a marked divergence in type 17 immunity between psoriasis and HS. HS is characterized by expansion of *IL17A*^+^*IL17F*^+^ T17 cells with an IL-1–associated inflammatory program and inflammatory remodeling of Tregs, rather than enrichment of IL-23-responsive *IL17A*^+^*IFNG*^+^ T17 cells observed in psoriasis. This disease-specific immune architecture provides a hypothesis-generating framework for potentially different responses to pathway-targeted therapies and motivates further evaluation of therapeutic approaches in HS, including dual IL-17A/IL-17F blockade and IL-1-targeted approaches.

## 1 Introduction

IL-17-producing Type 17 T-cells (T17) play a critical role in both psoriasis and hidradenitis suppurativa (HS), as supported by FDA approval of IL-17-blocking monoclonal antibodies (secukinumab and bimekizumab) for both conditions (1–4). However, clinical improvement with IL-17 blockers can be achieved in less than 55% of HS patients, while more than 80% of psoriasis patients achieve clinical remission with the same IL-17 blockers (5–7). These discrepancies suggest fundamental differences in the biology of T17 cells across these diseases.

We previously suggested that the less optimal clinical response with IL-17 blockers in HS compared to psoriasis is due to more diverse T17 cell activation pathways in HS than in psoriasis (8). Unlike psoriasis T17 cells, which are predominantly activated by IL-23, HS T17 cells are activated by a more diverse range of cytokines, including IL-1α, IL-1β, and IL-6 (2, 8). However, our previous single-cell comparison of psoriasis and HS was performed at the level of overall T17 cell cluster, without dissecting T17 cells at their subset levels.

Previous single-cell analyses from our group established a cytokine-combination framework in which T17 states are defined by the expression of IL17A, IL17F, IFNG, and IL10 (9). In a subsequent psoriasis study, we reported that psoriasis T17 cells have pathogenic versus non-pathogenic (protective/regulatory) T17 subsets in human skin, and they react differently to monoclonal antibodies blocking IL-23 (10). IL-23 inhibition selectively eliminates the likely pathogenic T17 subsets (specifically *IL17A*^+^*IFNG*^+^ and *IL17F*^+^*IL10*^-^ T17 subsets) while sparing *IL17A*^+^*IL17F*^+^ T17 cells, which remain as resident-memory cells in resolved psoriasis lesions and may primarily function in protective immunity (10). Whether analogous T17 subset programs exist in HS and how they differ from psoriasis remained incompletely defined. We therefore applied this prespecified framework to a cross-sectional comparison of psoriasis and HS, with atopic dermatitis (AD) included as a non–Type 17-dominant inflammatory skin disease.

## 2 Materials and methods

### 2.1 Data collection, preprocessing, and quality control

To compare single-cell transcriptomes of psoriasis, hidradenitis suppurativa (HS), atopic dermatitis (AD), and healthy control skin, we integrated our previously published psoriasis, HS, and healthy control skin single-cell RNA sequencing (scRNA-seq) datasets (8, 9) (GSE151177 and GSE220116) with a published collaborative dataset of AD and healthy control skin (11) (GSE147424). The study was designed as a cross-sectional comparison of lesional psoriasis and lesional HS skin with healthy control skin; matched non-lesional skin was not available across the included cohorts and was not analyzed. All eight HS libraries were derived from biologic-naive patients with Hurley stage III disease and were generated from tunnel-containing lesional tissue collected during surgical deroofing procedures performed by the authors of this study.

In total, 51 single-cell libraries (20 psoriasis, 8 HS, 4 AD, and 19 healthy control skin) were processed and integrated using the Seurat package (version 5.1.0) in R (version 4.3.3). Raw UMI count matrices were converted into individual Seurat objects after excluding cells expressing fewer than 100 or more than 5,000 detected genes, or cells with mitochondrial gene content exceeding 25%, to remove low-quality cells and potential doublets, consistent with the original source workflows.

### 2.2 Group-wise normalization and batch-aware feature selection

To facilitate batch-aware preprocessing and robust integration of single-cell data, samples were grouped based on three key technical factors: the originating study, the cell dissociation method used during sample preparation, and the version of 10x Genomics reagents applied. To minimize batch effects inherent to each of these technical variations, each group, defined by a unique combination of these factors, was independently log-normalized, and 2,000 highly variable features were selected using the variance-stabilizing transformation method. The normalized groups were then merged into a single dataset for joint integration and clustering. This group-wise normalization strategy was critical for preserving biological signals while reducing unwanted technical variation during data integration.

### 2.3 Dataset integration and batch-effect correction

Following the initial group-wise normalization and feature selection, the merged dataset was normalized to ensure consistency across all cells. Highly variable features were recomputed to capture key sources of biological variation. The data were then scaled using ScaleData(), followed by principal component analysis via RunPCA() to summarize the dataset’s variability.

To correct for batch effects and harmonize datasets originating from distinct technical conditions, we performed integration using IntegrateLayers() with the CCAIntegration method. This canonical correlation analysis (CCA)-based approach enables the alignment of shared biological signals across batches while adjusting for technical artifacts introduced by differences in dissociation protocols or reagent chemistry (12). Integration parameters (e.g., resolution and number of dimensions) were tuned to balance the removal of batch-specific variance while retaining biological heterogeneity. To evaluate potential overcorrection or loss of biological structure, we systematically compared clustering results, UMAP embeddings, and marker gene expression before and after integration.

### 2.4 Dimensionality reduction and clustering

Dimensionality reduction and clustering were subsequently performed on the integrated dataset to identify transcriptionally distinct cell populations. Shared nearest-neighbor (SNN) graphs were constructed using FindNeighbors() with the top 30 principal components derived from the integrated PCA space. Clusters were identified using FindClusters() with resolution values ranging from 0.1 to 0.4 to optimize the granularity of cluster separation. A resolution of 0.2 was selected for downstream analysis based on cluster stability and biological interpretability, assessed by cluster-specific marker gene expression and correspondence with known cell types.

### 2.5 Visualization and cell-type annotation

UMAP embedding was generated using RunUMAP() with the same 30 principal components to ensure consistency with the clustering results. Finally, JoinLayers() was used to merge the integrated dimensionality reduction outputs and metadata into a unified main assay object, enabling downstream analyses including marker gene detection and cell-type visualization. Cell-type annotation was conducted manually based on well-established canonical marker gene expression patterns in clusters defined at a resolution of 0.2.

### 2.6 Dual immunohistochemistry

Dual chromogenic immunohistochemistry was performed on HS lesional tissue sections to evaluate protein co-localization of nuclear FOXP3 with IL-1β, IL-17A, or IL-17F. Primary antibodies included anti-FOXP3 (Abcam; cat. no. ab20034), anti-IL-17A (Thermo Fisher Scientific; cat. no. MA5-51205), anti-IL-17F (Thermo Fisher Scientific; cat. no. MA5-63114), and anti-IL-1β (Novus Biologicals; cat. no. NBP1-42767). Sections were stained sequentially using species-specific secondary detection systems. FOXP3 was detected using the VECTASTAIN Elite ABC-HRP Anti-Mouse IgG Kit (Vector Laboratories; cat. no. PK-6102) followed by AEC substrate (Vector Laboratories; cat. no. SK-4200), producing a red chromogenic signal. IL-1β, IL-17A, and IL-17F were detected using the ImmPRESS-AP Horse Anti-Rabbit IgG Polymer Detection Kit (Vector Laboratories; cat. no. MP-5401-NB) followed by Vector Blue alkaline phosphatase substrate (Vector Laboratories; cat. no. SK-5300), producing a blue chromogenic signal. Double-positive cells were defined as cellular profiles exhibiting red nuclear FOXP3 staining together with blue cytokine staining associated with the same cell.

### 2.7 Statistical analysis

Differences in the proportion of each immune cell subset per sample among psoriasis, HS, AD, and healthy control skin were evaluated using the Kruskal-Wallis test, followed by Dunn’s multiple comparison test, as implemented in GraphPad Prism. For each sample, cell proportions were defined as the percentage of cells within a given immune cell subset that expressed the gene of interest. A p-value less than 0.05 was considered statistically significant.

## 3 Results

### 3.1 Cross-disease comparison identifies distinct T-cell, myeloid, and B-cell transcriptional features in HS

**Figure 1:**
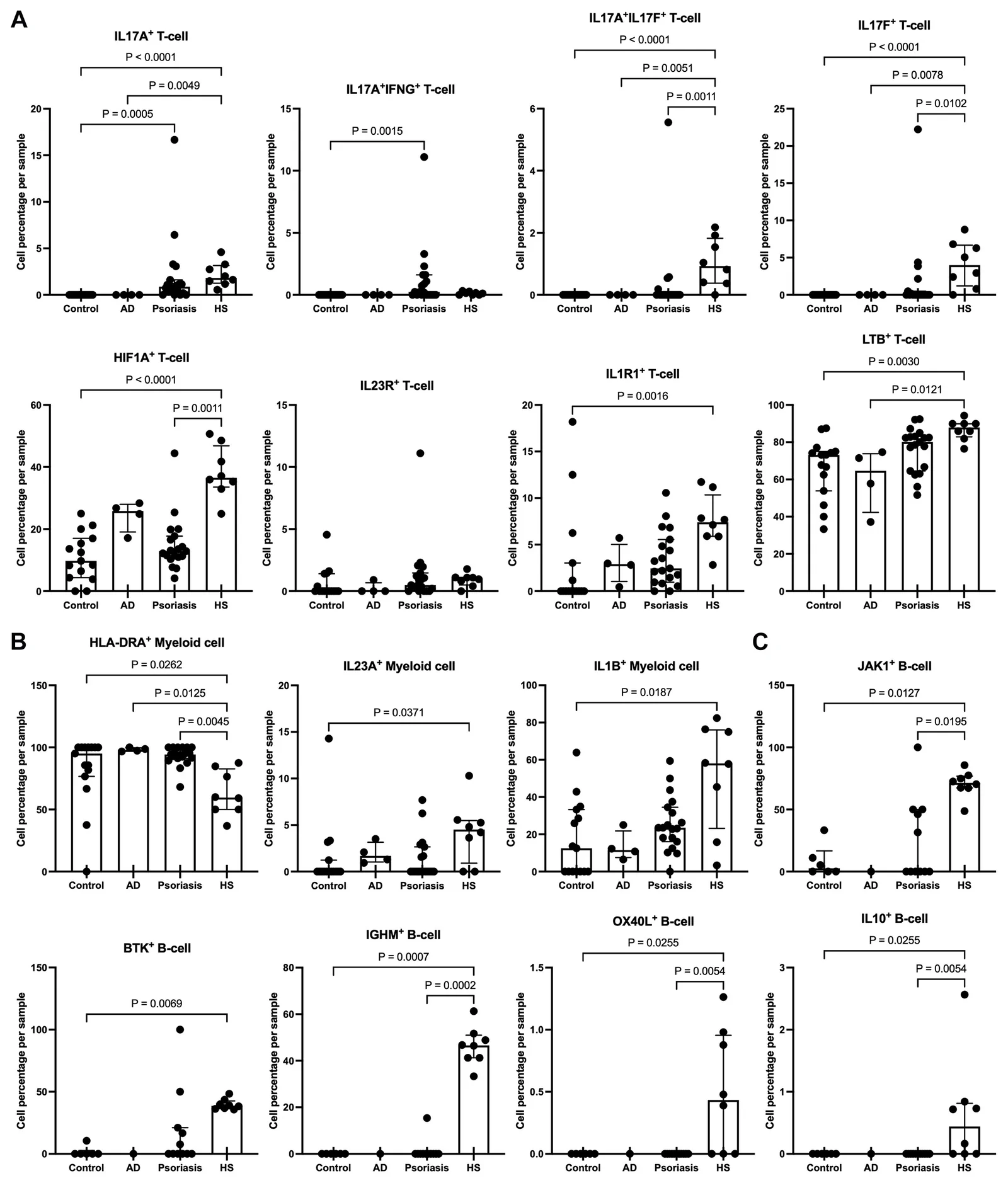
Frequency of immune cells expressing specific genes in control, atopic dermatitis (AD), psoriasis, and hidradenitis suppurativa (HS) lesional skin. Each dot represents the percentage of **(A)** T-cells, **(B)** myeloid cells, and **(C)** B-cells expressing the indicated genes in each sample.

We integrated scRNA-seq datasets from lesional skin of psoriasis (n=20), HS (n=8), AD (n=4), and healthy controls (n=19) and quantified the percentage of immune cells expressing selected transcripts per sample (**Figure 1**).

The percentage of T-cells expressing *IL17A* was significantly higher in both psoriasis and HS compared to control (p = 0.0005 and p < 0.0001, respectively) but not in AD (**Figure 1A**). Notably, *IL17A*^+^ *IFNG*^+^ T-cells, the pathogenic T17 subset in psoriasis (9, 10), were increased only in psoriasis (p = 0.0015), whereas HS lesions exhibited a higher frequency of *IL17F*^+^ T-cells and *IL17A*^+^ *IL17F*^+^ T-cells compared to controls (p < 0.0001) and even to psoriasis (p = 0.0102 and p = 0.0011, respectively). Although *IL23R*^+^ T-cell frequencies were comparable across the four conditions, an increased proportion of T-cells expressing *HIF1A* was observed only in HS lesions compared to controls (p < 0.0001). HS also showed increased frequencies of *IL1R1*^+^ T-cells compared with controls (p = 0.0016) and increased *LTB*^+^ T-cells compared with controls (p = 0.0030) and psoriasis (p = 0.0121).

Within myeloid cells, the proportion of *HLA-DRA*-expressing cells was reduced in HS compared with controls (p = 0.0262), AD (p = 0.0045), and psoriasis (p = 0.0125) (**Figure 1B**). Conversely, HS samples showed increased frequencies of *IL23A*^+^ myeloid cells compared with controls (p = 0.0371) and increased *IL1B*^+^ myeloid cells compared with controls (p = 0.0187).

Within B-cells, HS samples exhibited increased frequencies of *BTK*^+^ B-cells compared with controls (p = 0.0069) and increased *IGHM*^+^ B-cells compared with controls (p = 0.0007) and psoriasis (p = 0.0002) (**Figure 1C**). *JAK1*^+^ B-cells and *OX40L*^+^ B-cells were also increased in HS compared with controls (p = 0.0127 and p = 0.0255, respectively) and compared with psoriasis (p = 0.0195 and p = 0.0054, respectively). In addition, *IL10*^+^ B-cells were increased in HS compared with controls (p = 0.0255) and psoriasis (p = 0.0054).

### 3.2 T17 subset composition differs substantially between psoriasis and HS

**Figure 2:**
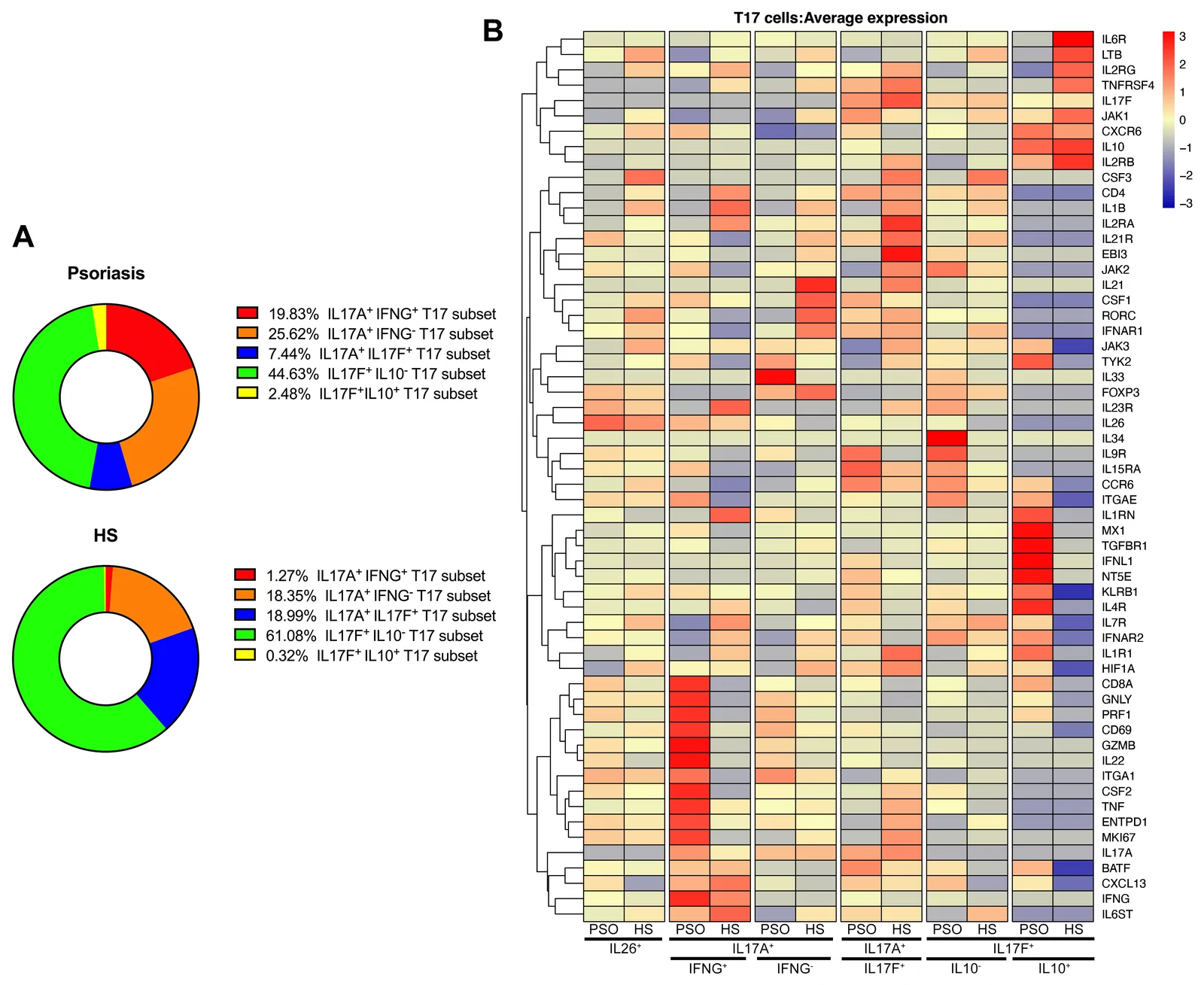
Single-cell transcriptome of Type-17 T-cell (T17) subsets in psoriasis and hidradenitis suppurativa (HS). **(A)** The proportion of T17 subsets in psoriasis and HS lesional skin. **(B)** Heatmap showing the average gene expression of T17 intermediates (*IL26*^+^*IL17A*^-^*IL17F*^-^ T17 cells; *IL26*^+^ T17) and five distinct T17 subsets in psoriasis (PSO) and HS lesional skin.

We next focused on T17 cells and subdivided them into five previously defined T17 subsets based on cytokine-expression (9, 10). Remarkably, the relative composition of these subsets differed between psoriasis and HS (**Figure 2A**). The *IL17A*^+^*IFNG*^+^ T17 subset, a pathogenic subset in psoriasis, comprised 19.83% of T17 cells in psoriasis lesional skin, while it was merely 1.27% in HS lesional skin. In contrast, the *IL17F*^+^*IL10*^-^ T17 and *IL17A*^+^*IL17F*^+^ T17 subsets were more abundant in HS compared to psoriasis (61.08% vs 44.63% and 18.99% vs 7.44%, respectively). Conversely, the *IL17F*^+^*IL10*^+^ T17, considered a regulatory T17 subset in psoriasis, was less prevalent in HS (0.32% vs 2.48%).

### 3.3 Activated inflammatory gene programs characterize the HS *IL17A*^+^*IL17F*^+^ T17 subset

Next, we compared average gene expression across T17 subsets between psoriasis and HS (**Figure 2B**). Consistent with previous studies (9, 10), the *IL17A*^+^*IFNG*^+^ T17 subset in psoriasis showed upregulation of cytotoxic transcripts, including *CD8A*, *GZMB*, *GNLY*, and *PRF1*. In contrast, this subset in HS exhibited a strikingly lower expression level of these cytotoxic transcripts, along with reduced expression of human tissue-resident memory T-cell (TRM) markers (13) (*CXCR6*, *CD103* (*ITGAE*), *CD69*, and *ITGA1*). Conversely, HS *IL17A*^+^*IFNG*^+^ T17 cells displayed elevated expression of the B-cell chemoattractant *CXCL13*.

Unexpectedly, the *IL17A*^+^*IL17F*^+^ T17 subset, which has been described as protective non-pathogenic population in psoriasis, exhibited a markedly increased pro-inflammatory transcriptional profile in HS. Compared with the same subset in psoriasis, HS *IL17A*^+^*IL17F*^+^ T17 cells expressed high levels of T17 cytokines (10, 14), including *TNF*, *CSF2*, and *IL21*. In addition, expression of inflammatory mediators such as *IL1B* and *CSF3* was increased. This subset also demonstrated elevated expression of *IL2RA* and *MKI67* in HS relative to psoriasis. Importantly, *IL1R1* and *TNFRSF4* (OX40) expression was higher in HS *IL17A*^+^*IL17F*^+^ T17 cells compared with their psoriasis counterparts.

### 3.4 Tregs in psoriasis and HS exhibit disease-specific inflammatory transcriptional remodeling

**Figure 3:**
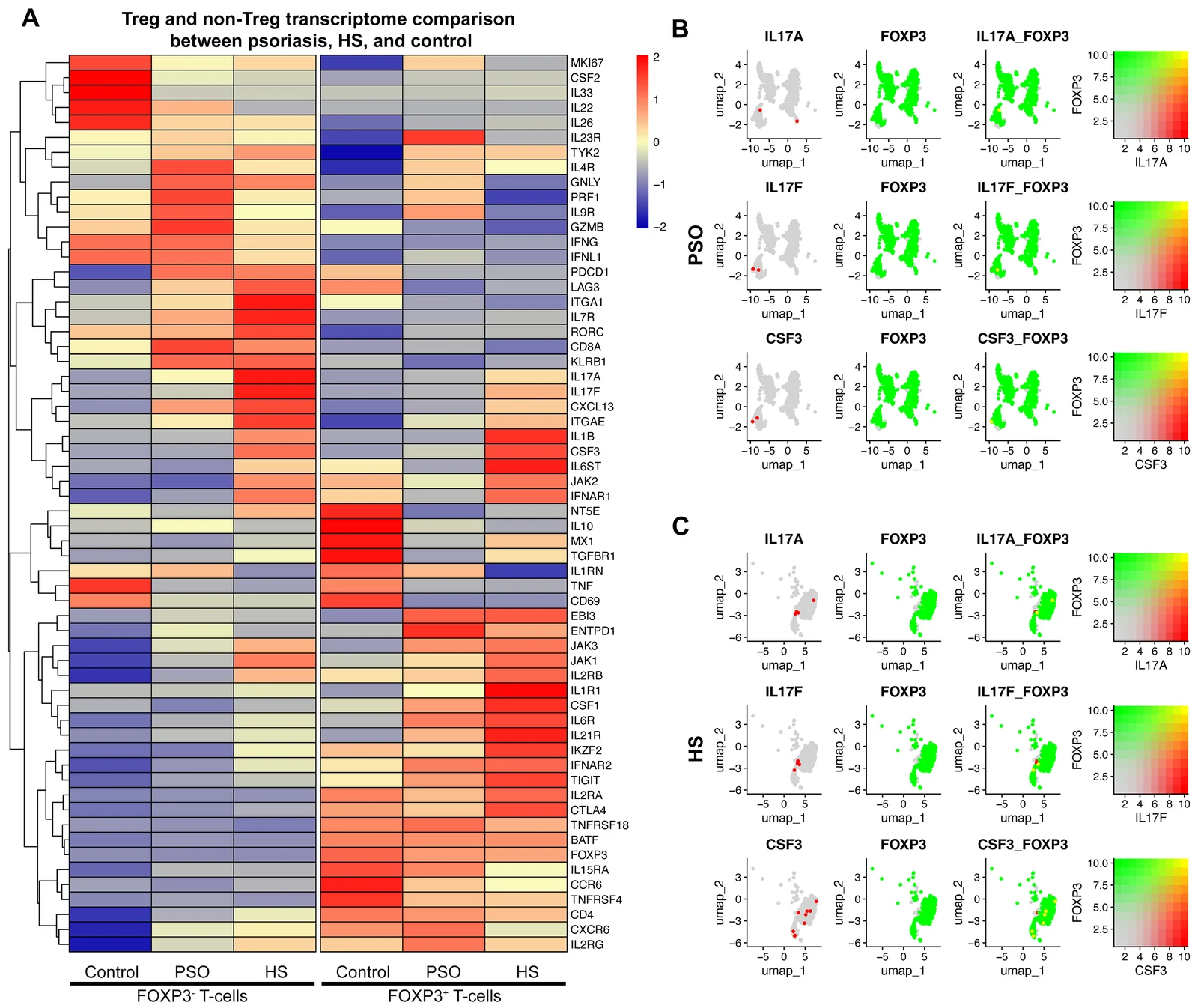
Single-cell transcriptome of regulatory T-cells in psoriasis and hidradenitis suppurativa (HS). **(A)** Heatmap showing the average gene expression of *FOXP3*^+^ and *FOXP3*^-^ T-cells in control, psoriasis (PSO), and HS lesional skin. **(B, C)** UMAP plots illustrating cytokine expression (left), *FOXP3* expression (middle), and co-expression of *FOXP3* with *IL17A*, *IL17F*, or CSF3 (right) in lesional PSO **(B)** and HS **(C)** *FOXP3*^+^ T-cells. Density plots depict co-expression intensities between *FOXP3* and each cytokine.

As the imbalance between T17 cells and regulatory T-cells (Tregs) is implicated in both psoriasis (15) and HS (16), we also investigated Tregs and compared their transcriptional profiles between psoriasis and HS (**Figure 3A**). Tregs were defined as *FOXP3*-expressing cells within T-cell clusters. *FOXP3*^+^ T-cells reliably represented the Treg population, exhibiting high expression of *FOXP3* and canonical Treg surface markers (17), *IL2RA*, *TNFRSF18*, and *CTLA4*, as well as low expression of *IL7R* compared to *FOXP3*^-^ T-cells.

In healthy control skin, *FOXP3*^+^ T-cells expressed high levels of the potent immunosuppressive cytokine *IL10.* In contrast, *FOXP3*^+^ T-cells from psoriasis and HS skin showed markedly reduced *IL10* expression, along with lower levels of *PDCD1, LAG3, and NT5E*, molecules associated with Treg induction and suppressive function (18–20). While IL10 expression persisted at a low level in psoriasis *FOXP3*^+^ T-cells, it was absent in HS (**Figures 3B, C**).

Furthermore, psoriasis *FOXP3*^+^ T-cells displayed increased expression of cytotoxic genes (*GNLY* and *PRF1*) and *IL23R*, whereas HS *FOXP3*^+^ T-cells were enriched for pro-inflammatory cytokines (*IL17A*, *IL17F*, *CXCL13*, *IL1B*, and *CSF3*), together with *IL1R1* (**Figures 3A, C**).

To assess whether the transcriptional co-expression patterns were also observable at the protein level, we performed dual immunohistochemistry on HS lesional tissue (**Supplementary Figure 1**). Cells showing nuclear FOXP3 staining together with IL-1β, IL-17A, or IL-17F protein staining were identified in HS lesions. These findings provide orthogonal evidence of protein co-localization but do not establish altered suppressive function, lineage conversion, or direct pathogenicity.

### 3.5 Murine Th17 gene signatures map differentially onto human cutaneous T17 subsets in psoriasis and HS

In addition to psoriasis and HS, T17 cells are key mediators of pathogenesis in various autoimmune diseases, including multiple sclerosis (MS), rheumatoid arthritis, and inflammatory bowel disease (21). Given the distinct inflammatory transcript profiles of T17 subsets observed in psoriasis and HS skin, we examined how previously defined murine pathogenic and non-pathogenic Th17 gene sets mapped onto these human cutaneous states. These gene sets were derived from *in vitro*-differentiated murine Th17 cells (22) and were subsequently linked to Th17 programs in experimental autoimmune encephalomyelitis and to CD4-positive T-cells from the cerebrospinal fluid of patients with MS (21).

**Figure 4:**
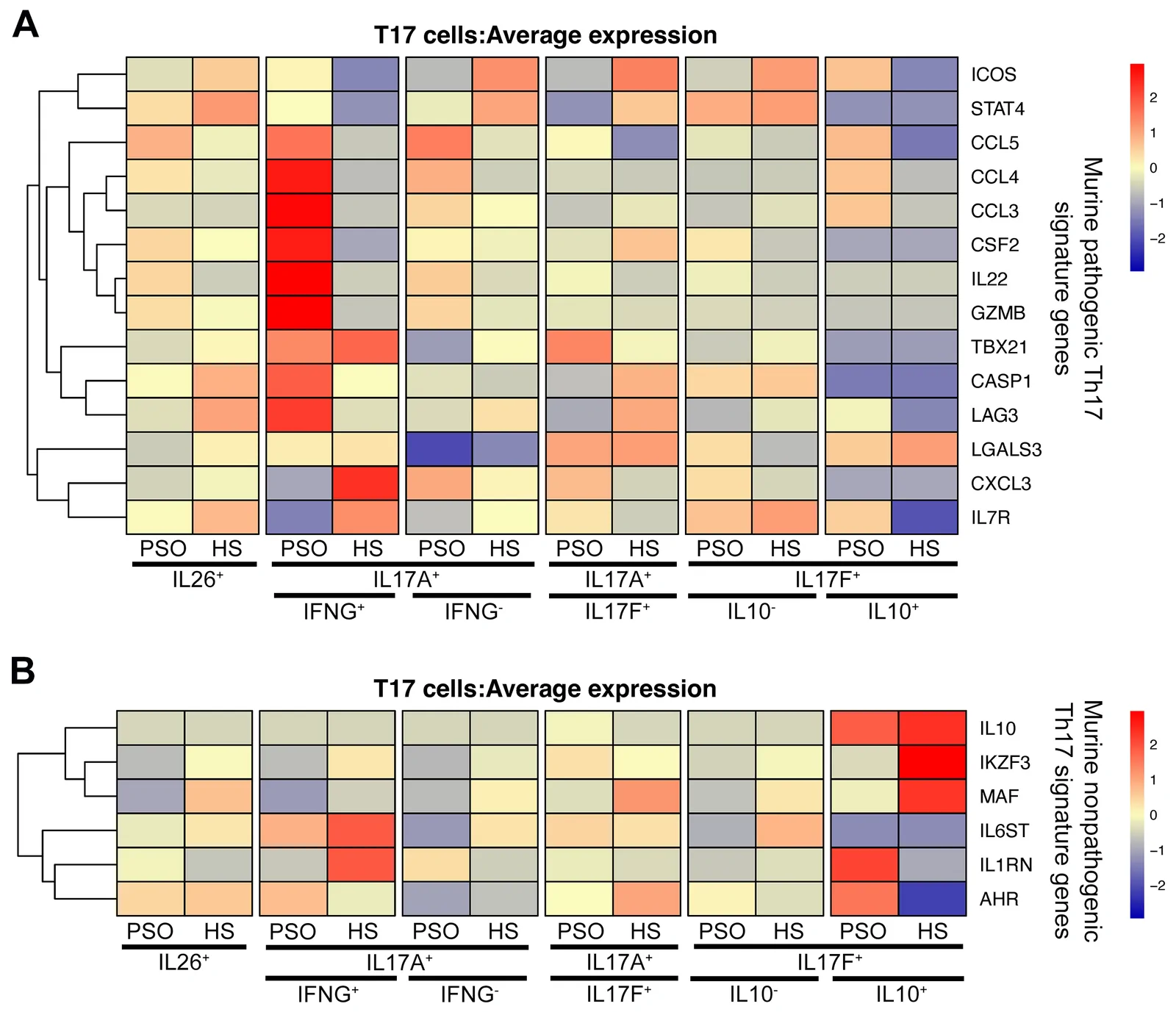
Differential expression of pathogenic and non-pathogenic murine Th17 signature genes in T17 subsets from psoriasis and hidradenitis suppurativa (HS). **(A)** Heatmap showing average expression of murine pathogenic Th17 signature genes in human T17 subsets from psoriasis (PSO) and HS lesional skin. **(B)** Heatmap showing average expression of murine non-pathogenic Th17 signature genes in the same T17 subsets. Gene sets were adapted from Lee et al., 2012, with murine genes lacking human orthologs excluded.

We therefore investigated the expression of these gene sets in our scRNA-seq dataset of T17 subsets from human psoriasis and HS skin. The expression pattern of murine pathogenic Th17 signature genes differed substantially between psoriasis and HS subsets (**Figure 4A**). In psoriasis, the *IL17A*^+^*IFNG*^+^ pathogenic subset exhibited high expression of multiple murine pathogenic signature genes, including *CCL5*, *CCL4*, *CCL3*, *CSF2*, *IL22*, *GZMB*, *CASP1*, *TBX21*, and *LAG3*. In contrast, expression of these genes was more heterogeneous across T17 subsets in HS. Expression of murine non-pathogenic Th17 signature genes also varied by disease and subset (**Figure 4B**). The *IL17F*^+^*IL10*^+^ T17 population in HS showed enriched expression of *IL10*, *IKZF3*, and *MAF* compared with other T17 subsets. These findings further support disease-specific organization of T17 transcriptional programs.

## 4 Discussion

Using a prespecified framework based on the combinatorial expression of *IL17A*, *IL17F*, *IFNG*, and *IL10*, we directly compared identically defined T17 states in psoriasis and HS. The central contribution of this study is the demonstration that the same cytokine-defined T17 subsets have markedly different distributions and transcriptional programs across two Type 17-mediated skin diseases. While both psoriasis and HS lesions showed increased *IL17A*^+^ T-cells compared with controls, the *IL17A*^+^*IFNG*^+^ T17 subset—previously associated with pathogenic, cytotoxic/TRM-like features in psoriasis (9, 10) —was largely absent in HS (**Figure 2A**). Instead, HS lesions were characterized by expansion of *IL17A*^+^*IL17F*^+^ T-cells with inflammatory and proliferative programs. These findings support the concept that the inflammatory potential of T17 states is disease context dependent.

The relative paucity of the IL17A^+^IFNG^+^ state in HS is consistent with, but does not establish, a different degree of IL-23 dependence between HS and psoriasis. In psoriasis, the IL-23/IL-17 axis is amplified by keratinocyte responses, and IL-23 blockade can selectively dampen pathogenic T17 populations, including *IL17A*^+^*IFNG*^+^ T17 cells and *IL17F^+^IL10^-^* T17 cells expressing *IL23R* (1, 10). In HS, although *IL23A* expression was increased in myeloid cells, the previously described IL-23 inhibitor-responsive *IL17A*^+^*IFNG*^+^ T17 subset was substantially decreased compared to psoriasis (19.83% vs 1.27%; **Figure 2A**). Clinical trial experience similarly suggests limited and inconsistent efficacy of IL-23-selective strategies in HS: a phase II trial of guselkumab did not meet its primary endpoint (23), and a phase II trial of risankizumab was terminated early with similar improvements across active and placebo arms (24). Taken together, these observations generate the hypothesis that the organization of IL-23-responsive T17 immunity differs between the diseases. However, the patients included in the present analysis were not prospectively followed for treatment response, and these data cannot establish a causal mechanism of drug response or a predictive biomarker.

In contrast, the *IL17A*^+^*IL17F*^+^ subset in HS showed a highly activated state, characterized by elevated *IL2RA* expression and expression of the proliferation-associated marker *MKI67*, along with co-expression of multiple inflammatory mediators including *TNF, CSF2, IL21, IL1B*, and *CSF3* (**Figure 2B**). *CSF3* is a critical regulator of neutrophil production and function (25), and neutrophils are a well-recognized component of HS pathology (26, 27). Immunostaining of lesional tissue demonstrated abundant G-CSF (CSF3) expression in HS, spatially associated with T-cell-rich areas, whereas no detectable staining was observed in psoriasis (**Supplementary Figure 2**). Although the *IL17A*^+^*IL17F*^+^ T17 subset has been proposed to represent a relatively non-pathogenic or homeostatic population in psoriasis, its quantitative expansion and inflammatory co-expression profile in HS support the concept of context-dependent T17-state biology. However, its direct pathogenic activity requires further investigation.

This subset also showed increased *IL1R1* expression in HS, raising the possibility that IL-1β may represent an important upstream signal for this population, potentially operating independently of IL-23. This interpretation is consistent with the recent study by Rastrick et al., which demonstrated that IL-1β promotes IL-17F expression independently of IL-23 (2). IL-1 signaling has long been implicated in HS pathogenesis and has shown therapeutic promise: anakinra (IL-1 receptor antagonist) demonstrated clinical benefit in a randomized trial (28), and IL-1α blockade with bermekimab showed improvement of inflammatory lesions and pain in a phase II study (29). In addition, dual neutralization of IL-1α and IL-1β with lutikizumab (ABT-981) has been evaluated in phase II studies in moderate-to-severe HS, with *post hoc* analyses reporting numerical reductions in draining tunnel counts compared with placebo (30). These observations support further investigation of IL-1 signaling as a potential upstream regulator of this T17 subset and warrant direct functional comparison with IL-23-dependent pathways.

Elevated *TNFRSF4* (OX40) expression in this inflammatory T17 subset suggests that OX40-mediated costimulatory signaling may contribute to HS inflammation and provides a rationale for investigating OX40-targeted therapies, which are already being evaluated in AD. Notably, this subset also expressed *TNF*, further supporting a highly activated inflammatory phenotype. In this context, brivekimig, a bispecific nanobody targeting TNF and OX40L, is currently undergoing phase II clinical evaluation in HS. However, perturbation studies and treatment-linked tissue cohorts will be required to determine whether OX40/OX40L and TNF signaling contribute to disease persistence, therapeutic resistance, or clinical response.

The increased frequency of *HIF1A*^+^ T-cells in HS may also reflect disease-specific microenvironmental pressure (**Figure 1A**). *HIF1A* is a hypoxia-inducible transcription factor that promotes T17 gene expression through RORγt and can suppress regulatory T-cell development (31). Because the HS samples were obtained from deep, structurally remodeled tunnel-bearing lesions, local hypoxia and metabolic stress may favor this transcriptional program. The co-occurrence of *HIF1A* expression and highly activated *IL17A*^+^*IL17F*^+^ T17 cells in HS therefore provides a potential link between the remodeled tunnel microenvironment and effector T-cell programming, although tissue oxygenation and metabolic function were not directly measured.

Beyond T-cell intrinsic alterations, our findings suggest coordinated programs of tertiary lymphoid organization and B-cell activation in HS, consistent with previous studies. We observed increased *LTB* expression among HS T-cells (**Figure 1A**)*. LTB* encodes lymphotoxin β, which along with its receptor LTβR plays a critical role for development of tertiary lymphoid structures (TLS), a hallmark of late-stage HS immunopathology (32). We further identified elevated *CXCL13* expression within selective T17 (*IL17A*^+^*IFNG*^+^) and Treg populations in HS (**Figure 2B** and **3A**). T-cell-derived *CXCL13* has been shown to recruit B-cells and promote TLS formation in chronic inflammatory settings (33, 34). Prior studies of late-stage HS have identified *CXCL13*-expressing inflammatory fibroblasts as a major cell source contributing to B-cell recruitment and TLS formation (32, 35). Moreover, our recent spatial transcriptomic analysis of early-stage HS (Hurley I) demonstrated upregulation of *CXCL13* in the superficial dermis, with complementary single-cell transcriptomic and immunohistochemical analysis identifying T-cells as the predominant source of this chemokine in early stage (36). Thus, the present findings extend, rather than independently establish, the concept of coordinated T-cell/B-cell organization in HS.

In parallel, HS B-cells were enriched for *BTK, JAK1, IGHM, OX40L*, and *IL10* expression (**Figure 1C**), consistent with enhanced activation and altered signaling pathways in B-cells. These findings align with growing evidence that B-cell and TLS programs contribute to chronic HS immunopathology, including reports of sustained cutaneous B-cell activity within HS TLS (35) and neutrophil-derived BAFF support of B-cells in HS lesions (37). Of translational relevance, Hambly et al. reported that baseline B-cell activation and complement pathways were enriched in patients with severe HS who subsequently did not respond to adalimumab (38). Collectively, our findings support the presence of coordinated T-cell and B-cell networks in HS and motivate further investigation of lymphoid organization and B-cell function as complementary therapeutic targets alongside canonical cytokine pathways. However, whether specific B-cell states contribute to therapeutic resistance will require prospective single-cell studies with baseline and on-treatment sampling.

We also observed disease-associated inflammatory remodeling of the *FOXP3*^+^ T-cell compartment (**Figure 3**). *FOXP3*^+^ T-cells from both diseases showed reduced *IL10* and lower detection of immunoregulatory molecules including *PDCD1, LAG3*, and *NT5E* (CD73), which are important for Treg induction, IL-10-mediated suppressive activity, and adenosine-dependent immunoregulation, respectively (18–20). Notably, HS *FOXP3*^+^ T-cells showed increased expression of *IL17A/IL17F* and inflammatory mediators (*IL1B, CSF3*) together with *IL1R1* compared with psoriasis, suggesting a more inflammatory Treg transcriptional state in HS. Dual immunohistochemistry provided orthogonal evidence of nuclear FOXP3 staining together with IL-1β, IL-17A, or IL-17F protein staining in HS lesions (**Supplementary Figure 1**). However, co-localization does not demonstrate loss of suppressive function, stable lineage conversion, or direct pathogenicity. Functional suppression assays and lineage or epigenetic analyses will be required to determine whether these cells retain regulatory activity or represent transitional inflammatory states.

Comparison with murine pathogenic/non-pathogenic Th17 signatures further emphasized the disease-specific organization of T17 programs in human skin (**Figure 4**). The murine pathogenic signature derived from *in vitro* Th17 polarization (22) has been linked to Th17 transcriptional programs in EAE mouse models and to CD4^+^ T-cells from the cerebrospinal fluid of patients with multiple sclerosis (21). In our dataset, this pathogenic signature mapped most strongly to the *IL17A*^+^*IFNG*^+^ subset in psoriasis but was distributed across multiple T17 populations in HS. These findings indicate that pathogenic Th17 transcriptional programs defined in non-cutaneous murine models do not universally translate to human inflammatory skin diseases.

This study has several limitations. First, it was a retrospective, cross-sectional integration of previously generated datasets rather than a prospectively powered comparison, and the psoriasis and HS samples were imbalanced (n=20 and n=8, respectively). In addition, the analysis integrated datasets generated across studies with differing acquisition protocols, although batch-aware methods were implemented to mitigate technical effects. All HS specimens consisted of tunnel-bearing lesional tissue collected during deroofing from biologic-naive patients with Hurley stage III disease. This clinical homogeneity improves definition of the sampled phenotype but limits generalizability to Hurley stage I or II disease, non-tunnel lesions, and earlier inflammatory stages. Because matched non-lesional skin was not available, patient-intrinsic background cannot be separated from lesion-associated remodeling. Clinical metadata were not fully harmonized across source datasets, and the modest HS sample size precluded stable multivariable adjustment; residual clinical and technical confounding therefore remains. Gene detection in scRNA-seq may be influenced by transcript dropout and ambient RNA in inflamed tissue. The dual immunohistochemistry supports protein co-localization but does not provide functional validation. Finally, no sequenced patient was prospectively linked to treatment response. Causal, functional, temporal, and treatment-predictive conclusions will require suppression assays, lineage or epigenetic analyses, direct perturbation studies, and prospective treatment-linked cohorts with paired baseline and on-treatment sampling.

In summary, our integrated single-cell analysis delineates a transcriptional divergence in Type 17 immunity between psoriasis and HS. HS is characterized by expansion of *IL17A*^+^*IL17F*^+^ T17 populations with a highly activated, IL-1-associated program, accompanied by coordinated B-cell/TLS signatures and inflammatory remodeling of Treg transcriptional states. These observations provide a hypothesis-generating framework for disease-context-dependent Type 17 immunity and potentially different responses to pathway-targeted therapies, warranting prospective and functional validation.

## Data Availability

The single-cell RNA sequencing datasets analyzed in this study are publicly available in NCBI’s Gene Expression Omnibus (GEO). The psoriasis, hidradenitis suppurativa (HS), and healthy control skin datasets are accessible under GEO Series accession numbers GSE151177 and GSE220116. The atopic dermatitis (AD) and additional healthy control skin datasets are available under GEO Series accession number GSE147424.

## Supplementary material

Supplementary Figure 1 Dual immunohistochemical staining of FOXP3 and inflammatory cytokines in hidradenitis suppurativa lesional skin. Representative dual chromogenic immunohistochemical staining for FOXP3 with IL-17A (top), IL-17F (middle), or IL-1β (bottom) in hidradenitis suppurativa lesional skin. FOXP3 was visualized as red nuclear staining, whereas IL-17A, IL-17F, and IL-1β were visualized as blue staining. Numbered boxed regions in the overview images are shown at higher magnification and illustrate representative FOXP3−/cytokine+, FOXP3+/cytokine+, and FOXP3+/cytokine− staining patterns. Double-positive cells were defined as cellular profiles showing red nuclear FOXP3 staining together with blue cytokine staining associated with the same cell. Scale bars: 100 μm in the overview images and 20 μm in the enlarged images.

Supplementary Figure 2 G-CSF (CSF3) expression in hidradenitis suppurativa compared with psoriasis. Representative immunohistochemical staining for CD3 and G-CSF performed on serial sections of lesional skin from patients with psoriasis and hidradenitis suppurativa (HS). In psoriasis (top panels), scattered CD3^+^ T-cells are present, while G-CSF staining is minimal. In contrast, HS lesions (bottom panels) show prominent CD3^+^ T-cell infiltration. Corresponding serial sections demonstrate abundant G-CSF expression localized to regions enriched for inflammatory infiltrates, spatially overlapping with areas containing CD3^+^ T-cells. Scale bars: 500 μm (psoriasis) and 200 μm (HS).
